# Interpretable Machine Learning for Predicting Cefoperazone–Sulbactam-Associated Coagulation Abnormalities in Elderly Inpatients: A Dual-Center Retrospective Study

**DOI:** 10.3390/diagnostics16010103

**Published:** 2025-12-28

**Authors:** Yajing Li, Hongru Deng, Yongquan Gu

**Affiliations:** 1 Department of Vascular Surgery, Xuanwu Hospital, Capital Medical University, Beijing 100053, China; 2 Department of Vascular Surgery, Fu Xing Hospital, Capital Medical University (FXH-CMU), Beijing 100038, China

**Keywords:** cefoperazone–sulbactam, coagulation abnormality, elderly inpatients, machine learning, SHAP interpretability

## Abstract

**Background/Objectives**: Cefoperazone–sulbactam is frequently prescribed to older inpatients for severe infections but has been associated with coagulation abnormalities, particularly among individuals with malnutrition or hepatic dysfunction. Early identification of at-risk patients remains challenging. To develop and validate a clinically interpretable model for predicting cefoperazone–sulbactam-related coagulation abnormalities in elderly inpatients and to provide practical tools for bedside risk estimation. **Methods**: We conducted a retrospective, dual-center study of 485 patients aged ≥ 60 years treated at Fuxing Hospital and Xuanwu Hospital of Capital Medical University who received cefoperazone–sulbactam for ≥72 h. Baseline clinical and demographic variables were analyzed using univariate and multivariable logistic regression to identify independent risk factors. Ten supervised machine-learning models were trained and evaluated using area under the ROC curve (AUC), accuracy, sensitivity, specificity, precision, and F1-score. SHapley Additive exPlanations (SHAP) were applied to assess model interpretability. A nomogram was constructed from the final logistic regression model, and a web-based calculator was developed for clinical use. **Results**: Multivariable analysis identified age ≥ 75 years, hypoproteinemia, total parenteral nutrition, insomnia, and recent oral antibiotic use as independent predictors of coagulation abnormalities. Among machine-learning models, LightGBM achieved the best overall performance by AUC and balanced classification metrics. SHAP analyses provided individualized and global explanations of feature contributions, facilitating clinical interpretation. The nomogram and web calculator enable rapid, patient-specific risk estimation. **Conclusions**: An interpretable machine-learning approach, complemented by a nomogram and web calculator, accurately stratifies the risk of cefoperazone–sulbactam-induced coagulation abnormalities in elderly inpatients. These tools may support personalized risk evaluation and earlier preventive interventions in routine care. The web-based calculator facilitates rapid bedside risk estimation and may help guide earlier monitoring and preventive interventions in routine care.

## 1. Introduction

Cefoperazone–sulbactam, a commonly used β-lactam/β-lactamase inhibitor combination, is widely prescribed for severe infections in older inpatients, such as pneumonia, urinary tract infection, and intra-abdominal sepsis [1,2,3]. Due to its broad-spectrum antimicrobial activity and effectiveness against extended-spectrum β-lactamase-producing organisms, cefoperazone–sulbactam is a first-line empirical therapy in many tertiary care institutions. In older people who tend to have comorbidities, compromised immune systems, and prolonged hospitalizations, cefoperazone–sulbactam provides reliable coverage in cases of polymicrobial nosocomial infections [1,3,4]. Despite that, its wide application in older patients also calls for close surveillance of drug-associated adverse events, especially those that are insidious or not correctly estimated in the routine care [5,6,7].

Although clinically valuable, cefoperazone–sulbactam has been implicated in coagulation dysfunction, such as prolonged prothrombin time (PT) and activated partial thromboplastin time (APTT), as well as bleeding events. These complications often go underdiagnosed, especially in elderly patients with multiple comorbid conditions [8,9]. Possible mechanisms include suppression of synthesis of vitamin K–dependent clotting factors, possibly by the N-methylthiotetrazole (NMTT) side chain of cefoperazone, and alteration of intestinal flora, which diminishes the production of endogenous vitamin K. Older adults are at higher risk of being susceptible to these effects caused by age-related hepatic insufficiency, lowered dietary intake, reduced diversity of gut flora, and more commonly used broad-spectrum antibiotics, or antiplatelet drugs [2,9,10,11].

In a clinical setting, these alterations may be subtle or identified only in the setting of bleeding complications. Considering the high utilization rate of cefoperazone–sulbactam in geriatric medicine, it is clinically significant to take a proactive role in recognizing patients who carry a higher risk, which is currently lacking.

Although several case reports and pharmacovigilance studies have reported on coagulopathy related to cefoperazone, few attempts have been made to identify patients at the highest risk. Much of the literature to date has been univariate or has detected bleeds post hoc and, therefore, provides little indication for prospective clinical decision-making. However, there are no established methods for assessing coagulation risk before or while antimicrobial treatment is performed in older people [8,12,13].

This lack of risk stratification is particularly problematic in frail older patients, who frequently present with overlapping risk factors, i.e., malnutrition, polypharmacy, liver insufficiency, and insomnia, which together may enhance their vulnerability [14,15]. In the absence of predictive guidance, clinicians have to rely on their judgment, which could delay therapy or, conversely, result in continuation of treatment in low-risk subjects.

Because there were no established risk stratification systems for antibiotic-associated coagulopathy, the present study attempted to develop an easily applicable prediction model for detecting the development of coagulation abnormalities in elderly inpatients receiving treatment with cefoperazone–sulbactam. We first used multivariable logistic regression to identify independent clinical risk factors with retrospective data from two tertiary care hospitals. To further enhance predictive performance and explore algorithmic alternatives, we implemented and compared ten supervised machine learning models. Machine learning methods can model complex nonlinear relationships and interactions among clinical variables, which may improve risk stratification when multiple overlapping geriatric risk factors coexist. This complements regression-based tools by providing an algorithmic alternative when conventional models may be limited in capturing such complexity.

We also applied SHapley Additive exPlanations (SHAP) to enhance interpretability and provide individualized explanations of feature contributions. We constructed a nomogram for bedside use and developed a web-based tool to support real-time clinical decision-making. The aim of this integrative process was early identification of risk, therapeutic approach guide extension, belonging to the clinical admittance, and patient safety by antibiotics management.

## 2. Materials and Methods

### 2.1. Study Population and Data Collection

This was a retrospective observational study performed at 2 tertiary hospitals in Beijing, namely Fuxing Hospital and Xuanwu Hospital of Capital Medical University. Elderly inpatients (aged ≥ 60 years) who received intravenous cefoperazone–sulbactam treatment for at least 72 h between August 2020 and August 2024 were considered for inclusion. We clarified that ≥72 h is an inclusion criterion (minimum exposure), while outcome ascertainment spans hospitalization after initiation. Patients with pre-existing coagulation defects, concomitant anticoagulant treatment, and incomplete medical records were excluded. Pre-existing coagulation defects were defined as any documented congenital or acquired coagulation disorder (e.g., hemophilia, von Willebrand disease, disseminated intravascular coagulation, or advanced liver disease–related coagulopathy), or abnormal baseline coagulation tests prior to cefoperazone–sulbactam exposure, defined as PT or APTT > 1.5× the upper limit of normal.

The main outcome was coagulation impairment, defined as prolonged PT, APTT, or evidence of bleeding during hospitalization. The primary outcome (coagulation abnormality) was defined as PT > 18 s or >1.5× the upper limit of normal, and/or APTT > 60 s or >1.5× the upper limit of normal, occurring after cefoperazone–sulbactam initiation during hospitalization; clinically evident bleeding events documented in the medical record were also counted as outcome events. A total of 485 patients fulfilled the inclusion criteria. Clinical and demographic information was obtained from electronic medical record systems and consisted of 18 potential variables categorized as demographics, comorbidities, medication history, nutrition status, and lifestyle.

### 2.2. Data Preprocessing and Cohort Splitting

All categorical variables were converted to binary variables or dummy variables, and continuous variables were checked for normal distribution and standardized, when necessary. Continuous variables (primarily BMI) were standardized using z-score normalization (mean = 0, standard deviation = 1) calculated from the training set and applied to the test set. Because BMI in our cohort was approximately normally distributed, no transformation was required; if substantial skewness had been present, a log-transformation would have been considered prior to standardization. Missing values were handled using complete-case analysis (listwise deletion). Because no predictor had >5% missingness, no imputation was performed; records with missing predictor values were excluded from model training and evaluation. The dependent variable, coagulation abnormality status, was dichotomous (present/absent).

The dataset was randomly divided into training (70%) and test (30%) datasets with stratified sampling to maintain the distribution of the positive outcome for the training and test sets. The following definitions were uniform: age (60–75, ≥75 y); feeding_state (normal diet, transnasal gastric tube feeding, and total parenteral nutrition); hypoproteinemia (serum albumin < 35 g/L); and lifestyle factors [smoking, drinking, and insomnia (present or absent)]. These feature sets were applied to all subsequent modeling and interpretability analyses.

### 2.3. Logistic Regression and Feature Selection

Logistic regression was used to identify independent predictors of cefoperazone–sulbactam-related coagulopathy. Univariate logistic regression was performed on each potential variable in the training set. Variables showing a potential significant relationship with the outcome (*p* < 0.05) were entered in a multivariable logistic regression model. A backward stepwise elimination method was used to iteratively remove nonsignificant variables, thereby refining the model to include predictors that were statistically and clinically relevant.

The multivariable logistic regression model was used to calculate adjusted odds ratios (ORs) with 95% confidence intervals (CIs) to assess the independent contribution of each variable in relation to confounding. This method provided insight into independent clinical and demographic variables that may predict the risks of coagulation dysfunction by cefoperazone–sulbactam exposure. The predictor variables in the final model were then chosen as input predictors for subsequent machine learning model building and interpretability analysis.

### 2.4. Model Training and Validation by Machine Learning Model

We used ten supervised machine learning methods for building the prediction models of coagulation abnormalities, including AdaBoost, CatBoost, Gradient Boosting Machine (GBM), k-Nearest Neighbors (KNN), Light Gradient Boosting Machine (LightGBM), logistic regression, Neural Network, Random Forest, Support Vector Machine (SVM), and eXtreme Gradient Boosting (XGBoost). Input features were those originated from the variables included in the multivariable logistic regression, and each model was trained in the training data.

For optimization of performance and overfitting, model training and internal validation were performed with ten-fold cross-validation. Hyperparameter tuning was performed using grid search when applicable. Comparisons were made between the training set and test set’s model performance based on a set of metrics, including AUC, accuracy, sensitivity, specificity, precision, and F1 score. Discriminative power was measured with ROC curves, model calibration was assessed using calibration plots, and net clinical benefit was evaluated using decision curve analysis (DCA).

### 2.5. Model Interpretation, Nomogram, and Web Deployment

To enhance the interpretability and clinical transparency of the best-performing machine learning model, SHapley Additive exPlanations (SHAP) analysis was performed. SHAP values quantified each feature’s contribution to the prediction at both the global and individual levels. Visualization outputs included summary plots, dependence plots, force plots, and waterfall charts, which offered insight into how specific variables influenced model outputs.

Additionally, a nomogram was constructed based on the final multivariable logistic regression model to provide an intuitive, point-based clinical tool for individualized risk estimation. Nomograms were developed using both the full dataset and the training subset to assess robustness and generalizability. Finally, an interactive web-based prediction tool was built using the Shiny framework. This tool allows clinicians to input patient-specific information and obtain real-time risk estimates for cefoperazone–sulbactam-associated coagulation abnormalities. All processes were performed using R software (version 4.5.0).

## 3. Results

### 3.1. Baseline Characteristics of the Study Population

A total of 485 elderly inpatients were included in the study, comprising 335 patients without coagulation abnormalities and 150 patients who developed coagulation abnormalities following the administration of cefoperazone–sulbactam. The baseline characteristics of the two groups are summarized in Table 1. Patients in the coagulation abnormalities group were significantly older, with 61.3% aged ≥ 75 years compared to 42.4% in the non-abnormal group (*p* < 0.001). A higher proportion of patients in the abnormal group had a history of intravenous (40.7% vs. 27.5%, *p* = 0.005) and oral (56.7% vs. 38.5%, *p* < 0.001) antibiotic use. Nutritional support methods also differed markedly, with total parenteral nutrition (18.0% vs. 8.4%) and transnasal gastric tube feeding (44.7% vs. 37.9%) more prevalent in the abnormal group (*p* < 0.001). Hypoproteinemia was significantly more frequent among patients with coagulation abnormalities (88.0% vs. 66.6%, *p* < 0.001), as was the use of antiplatelet medications (43.3% vs. 31.3%, *p* = 0.014). Insomnia was also more commonly reported in this group (82.7% vs. 69.0%, *p* = 0.002). No significant differences were observed between the two groups in terms of gender, marital status, smoking, drinking, hypertension, diabetes, cardiovascular disease, hepatic or renal insufficiency, hyperlipidemia, or body mass index (all *p* > 0.05). These variables were retained for further modeling to assess their potential contributions to the risk of coagulation abnormalities.

### 3.2. Logistic Regression Analysis of Risk Factors

Univariate and multivariate logistic regression analyses were conducted to identify independent risk factors for cefoperazone–sulbactam-associated coagulation abnormalities in the training cohort. As shown in Table 2, several variables were significantly associated with coagulation risk in univariate models, including age ≥ 75 years (OR: 2.14, 95% CI: 1.34–3.43, *p* = 0.002), history of intravenous (OR: 2.14, *p* = 0.002) and oral (OR: 2.27, *p* < 0.001) antibiotic use, total parenteral nutrition (OR: 4.07, *p* < 0.001), antiplatelet use (OR: 1.99, *p* = 0.005), hypoproteinemia (OR: 2.33, *p* = 0.005), and insomnia (OR: 2.40, *p* = 0.003).

Multivariate logistic regression confirmed five independent predictors: age ≥ 75 years (adjusted OR: 2.13, 95% CI: 1.27–3.56, *p* = 0.004), oral antibiotic use (adjusted OR: 1.90, 95% CI: 1.13–3.19, *p* = 0.015), total parenteral nutrition (adjusted OR: 3.82, 95% CI: 1.71–8.53, *p* = 0.001), hypoproteinemia (adjusted OR: 3.19, 95% CI: 1.64–6.19, *p* < 0.001), and insomnia (adjusted OR: 2.94, 95% CI: 1.56–5.52, *p* < 0.001). These variables were retained as inputs for subsequent machine learning modeling due to their robust statistical significance and clinical relevance. Other variables, such as gender, cardiovascular disease, and antiplatelet use, lost statistical significance in multivariate analysis and were excluded from model training.

### 3.3. Performance of Machine Learning Models

**Figure 1:**
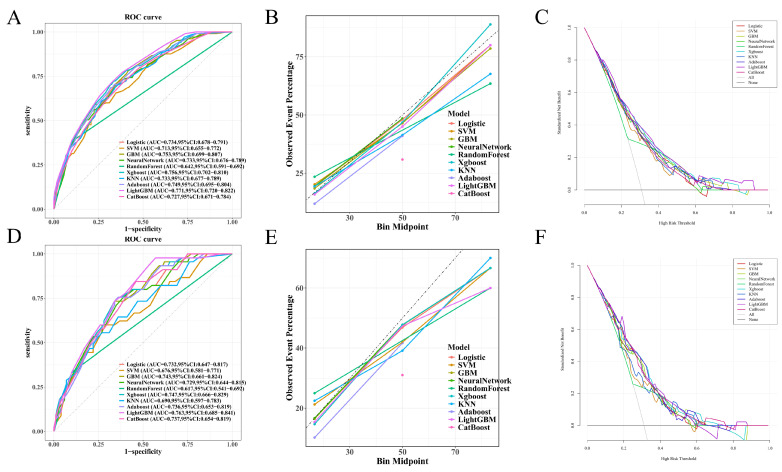
Performance of machine learning models in training and test sets: (**A**–**C**) In the training set, LightGBM achieved the highest AUC (0.771, 95% CI: 0.720–0.822). Calibration curves showed good agreement between predicted and observed outcomes, particularly for LightGBM, GBM, and XGBoost. Decision curve analysis (DCA) demonstrated that LightGBM yielded the greatest net clinical benefit across a wide range of threshold probabilities. (**D**–**F**) In the independent test set, LightGBM also achieved the highest AUC (0.763, 95% CI: 0.685–0.841), followed by XGBoost and GBM. Calibration and DCA curves further supported the superior predictive performance and clinical utility of LightGBM.

Ten machine learning algorithms were developed using predictors identified in the multivariate logistic regression model, and their performance was evaluated on both the training and test sets. As summarized in Table 3, the LightGBM model demonstrated the highest area under the receiver operating characteristic curve (AUC) in both datasets. In the test set, LightGBM achieved an AUC of 0.763 (95% CI: 0.685–0.841), followed closely by XGBoost (AUC: 0.747) and GBM (AUC: 0.743), as shown in Figure 1D. In the training set, LightGBM also achieved the highest AUC (0.771, 95% CI: 0.720–0.822), as illustrated in Figure 1A.

Calibration curves (Figure 1B,E) demonstrated that most models exhibited good agreement between predicted and observed risks, particularly LightGBM, GBM, and XGBoost. Decision curve analysis (DCA) (Figure 1C,F) further confirmed the clinical utility of these models, with LightGBM yielding the highest standardized net benefit across a broad range of threshold probabilities in both datasets.

In terms of other metrics, LightGBM in the test set exhibited the highest sensitivity (97.8%) and F1-score (0.603), despite moderate specificity (43.0%), indicating its superior ability to identify true positives. Models like Random Forest showed high specificity but suffered from low sensitivity and F1-score. Overall, LightGBM demonstrated the most balanced and robust performance and was thus selected for further interpretation and model deployment.

### 3.4. Confusion Matrix Analysis of Classification Performance

**Figure 2:**
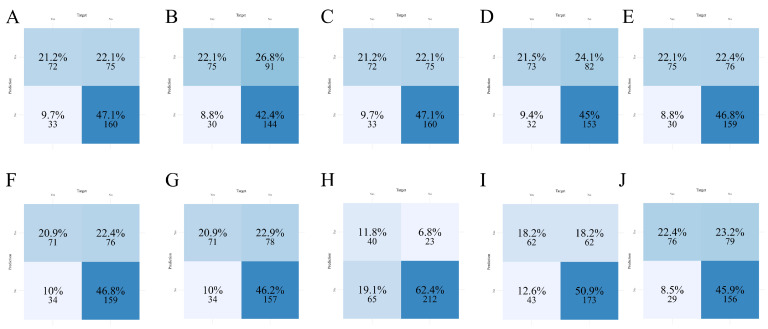
Confusion matrices of ten machine learning models in the training set. (**A**) AdaBoost; (**B**) CatBoost; (**C**) GBM; (**D**) KNN; (**E**) LightGBM; (**F**) Logistic Regression; (**G**) Neural Network; (**H**) Random Forest; (**I**) SVM; (**J**) XGBoost.

**Figure 3:**
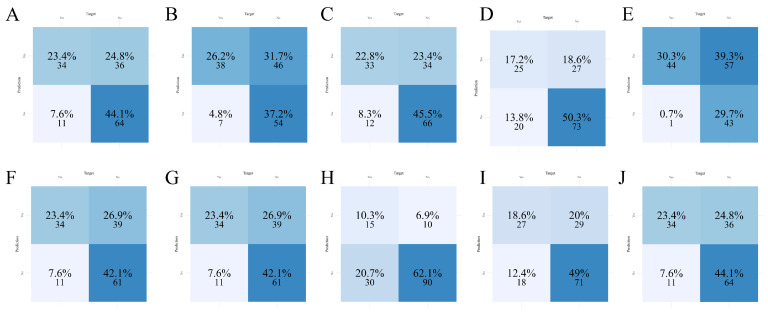
Confusion matrices of ten machine learning models in the test set. (**A**) AdaBoost; (**B**) CatBoost; (**C**) GBM; (**D**) KNN; (**E**) LightGBM; (**F**) Logistic Regression; (**G**) Neural Network; (**H**) Random Forest; (**I**) SVM; (**J**) XGBoost.

Confusion matrices for each of the ten machine learning models were generated to evaluate classification performance in both the training (Figure 2) and test (Figure 3) sets. In this setting, sensitivity was prioritized because failing to identify a truly high-risk patient may delay monitoring or preventive measures and increase the likelihood of clinically significant bleeding, while false-positive alerts primarily prompt closer surveillance with relatively low harm. In the training set, LightGBM (Figure 2E) demonstrated a favorable balance between true positives (TP) and true negatives (TN), with minimal false negatives (FN), highlighting its sensitivity in identifying patients at risk for coagulation abnormalities. GBM (Figure 2C) and XGBoost (Figure 2J) also showed high TP counts but had slightly increased false positive (FP) rates compared to LightGBM. Conversely, Random Forest (Figure 2H) and SVM (Figure 2I) exhibited more conservative behavior with higher TNs but lower TPs, suggesting reduced sensitivity.

In the test set, LightGBM again demonstrated dominant classification performance (Figure 3E), with the highest sensitivity as previously supported by Table 3, but at the expense of increased false positives. This trade-off suggests a prioritization of detecting abnormal cases over avoiding unnecessary alerts, which may be clinically acceptable in high-risk settings. Logistic Regression (Figure 3F) and Neural Network (Figure 3G) models yielded moderate classification balance, whereas models such as KNN (Figure 3D) and Random Forest (Figure 3H) underperformed in identifying positive cases. Overall, the matrix-based evaluations visually confirmed the superior detection capability of LightGBM, justifying its further exploration for interpretability and deployment.

### 3.5. Feature Importance and SHAP-Based Model Interpretation

**Figure 4:**
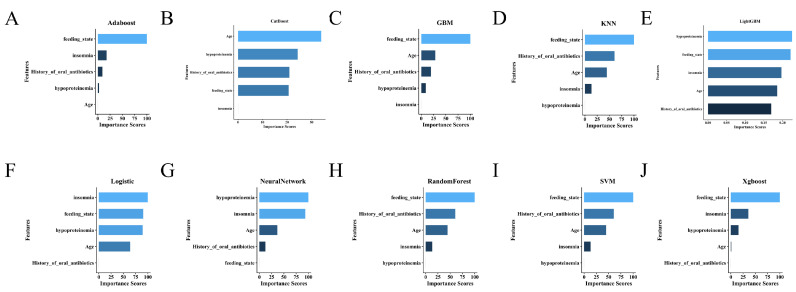
Comparison of feature importance across ten machine learning models. (**A**) AdaBoost; (**B**) CatBoost; (**C**) GBM; (**D**) KNN; (**E**) LightGBM; (**F**) Logistic Regression; (**G**) Neural Network; (**H**) Random Forest; (**I**) SVM; (**J**) XGBoost.

**Figure 5:**
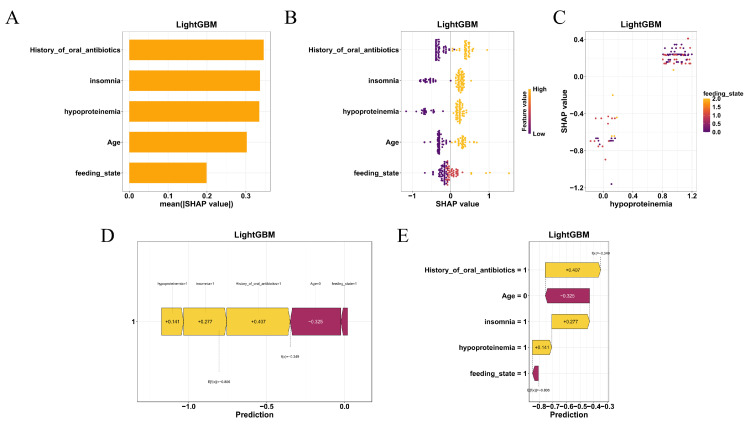
SHAP-based interpretability of the LightGBM model. (**A**) Mean absolute SHAP values showing the global impact of each feature. (**B**) SHAP beeswarm plot illustrating the direction and magnitude of individual feature effects. (**C**) SHAP dependence plots showing nonlinear interactions, between feeding state and hypoproteinemia. (**D**) SHAP force plot and (**E**) waterfall chart explaining individual patient predictions by visualizing how risk factors contribute to the model’s output.

To further elucidate the decision-making mechanisms across different models, feature importance analyses were conducted for all machine learning algorithms, with SHAP (SHapley Additive exPlanations) interpretation performed specifically for the best-performing model, LightGBM. In SHAP plots, positive values indicate that a feature increases the predicted risk relative to the baseline, whereas negative values indicate a decrease; larger absolute values reflect a stronger influence on the prediction. As shown in Figure 4, the top five most influential features included feeding_state, age, hypoproteinemia, insomnia, and history_of_oral_antibiotics. This ranking was further supported by the mean absolute SHAP values (Figure 5A), which quantified each variable’s contribution to model output.

The SHAP beeswarm plot (Figure 5B) illustrated the direction and magnitude of individual feature impacts on the predicted risk. Notably, hypoproteinemia and enteral feeding (tube or parenteral nutrition) consistently contributed to a higher probability of coagulation abnormalities, while younger age and absence of insomnia were associated with decreased risk. SHAP dependence plots (Figure 5C) revealed nonlinear interactions, particularly between feeding_state and hypoproteinemia, further emphasizing the model’s sensitivity to combinations of nutritional and clinical factors.

At the individual level, SHAP force plots (Figure 5D) and waterfall charts (Figure 5E) provided transparent visualizations of the prediction for specific patients. These interpretive visualizations delineated how combinations of high-risk features—such as older age, nutritional compromise, and prior antibiotic use—aggregated to shift the model’s output toward a positive prediction.

Together, the SHAP-based interpretability framework enhanced the clinical transparency of the LightGBM model and supported its applicability in individualized risk prediction.

### 3.6. Nomogram Construction for Individualized Risk Estimation

**Figure 6:**
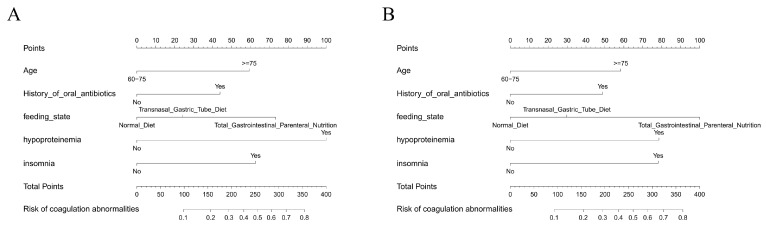
Nomograms for individualized risk prediction: (**A**) Nomogram based on the full dataset for estimating the risk of cefoperazone–sulbactam-associated coagulation abnormalities. (**B**) Nomogram derived from the training set, showing similar predictor structure and stability.

To facilitate clinical application, nomograms were constructed based on the final logistic regression model using the top predictors identified through multivariate analysis. As shown in Figure 6A, the nomogram incorporating the entire dataset provides a quantitative scoring tool for estimating the probability of cefoperazone–sulbactam-associated coagulation abnormalities in elderly patients. Each predictor—such as age, hypoproteinemia, insomnia, oral antibiotic use, and nutritional support—was assigned a weighted point scale reflecting its relative contribution to risk.

An additional nomogram was generated using only the training dataset (Figure 6B) to assess consistency and internal validity. The structure and weighting of predictors in both nomograms were largely concordant, indicating model stability across sampling subsets. These nomograms allow clinicians to compute individualized risk scores and visually interpret how combinations of patient factors translate into predicted risk.

### 3.7. Web-Based Risk Prediction Tool Deployment

**Figure 7:**
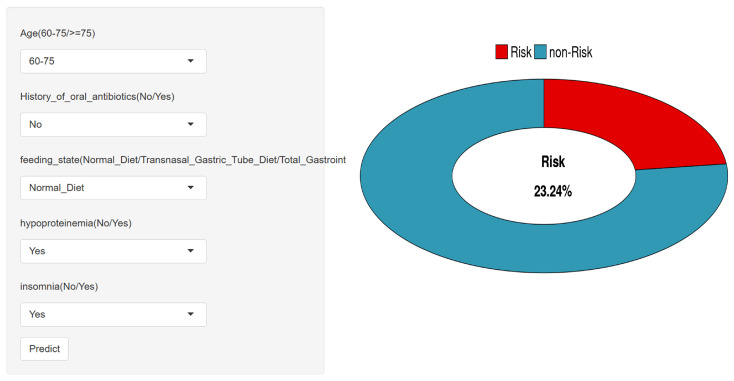
Web-based risk prediction tool interface for clinical use.

To enhance accessibility and promote clinical translation, a web-based risk prediction tool was developed using the Shiny platform. As illustrated in Figure 7, the interface allows users to input patient-specific variables—such as age group, nutritional status, presence of hypoproteinemia, insomnia, and recent oral antibiotic use—and instantly receive an individualized risk estimate for developing cefoperazone–sulbactam-associated coagulation abnormalities. The predictive engine is powered by the optimized LightGBM model, and the tool provides a user-friendly visualization of both probability output and contributing factors.

The application is publicly accessible at https://cacsriskmodel.shinyapps.io/make_web-1/ (29 November 2025), supporting shared decision-making and real-time clinical use. This tool serves as a practical extension of the study’s findings and reinforces the feasibility of integrating machine learning into bedside risk assessment.

## 4. Discussion

In this work, a predictive model was established and validated to identify the risk of coagulation abnormalities in elderly inpatients treated with cefoperazone–sulbactam. We initially employed multivariable logistic regression to determine potential independent risk factors based on routinely collected clinical variables. We then trained and tested ten machine learning models, and LightGBM achieved better discrimination in both the training and test sets. Additional predictive power beyond the model was provided by SHapley Additive exPlanations (SHAP) analysis, which provided interpretable and transparent observations of feature importance at the population and per-individual level. We further established a nomogram using logistic regression results in order to establish a simple, point-based risk prediction tool. For clinical relevance, a web-based application was created that enabled real-time personalized risk prediction. These results also imply that machine learning, especially LightGBM, may serve as a powerful tool for early alerting of cefoperazone–sulbactam-related coagulation risks in elderly patients.

A number of clinical factors were found to be independent risk factors for cefoperazone–sulbactam-associated coagulation abnormalities in older people, all appropriate to their pathophysiological mechanism. Older age (age ≥ 75 years) was also significantly associated with risk, which is likely to have been mediated by age-related hepatic metabolic dysfunction, reduced absorption of vitamin K, and the higher prevalence of subclinical nutritional deficiencies, all common among older adults and leading to coagulopathy [16,17].

Hypoproteinemia was also significantly correlated with abnormal coagulation. Serum albumin is responsible for the maintenance of oncotic pressure and transport of coagulation factors, and a decrease in serum albumin levels may be associated with malnutrition and impaired hepatic synthetic function, which can lead to increased bleeding tendency [18,19,20,21]. Additionally, total parenteral nutrition was identified as a risk factor, likely related to low enteral vitamin K intake and alterations in the gut microbiota, both of which may lead to derangement in vitamin K-dependent coagulation pathways [22,23].

Insomnia, being less traditionally associated with hemostatic outcomes, might be a marker of frailty, inflammation, or neuroendocrine dysregulation in older people, all of which have been previously associated with changes in platelet function and clotting cascades [24,25,26]. Insomnia should be interpreted cautiously as a clinical marker rather than a confirmed causal driver of antibiotic-associated coagulopathy. Sleep disturbance in older inpatients may reflect frailty, systemic inflammation, neuroendocrine dysregulation, or higher illness severity, each of which may correlate with hemostatic vulnerability. In addition, residual confounding is possible because detailed exposure to sedative–hypnotics, over-the-counter agents, and herbal supplements with potential antiplatelet effects (e.g., Ginkgo preparations) was not consistently captured in this retrospective dataset. Therefore, the observed association may partly represent unmeasured co-medication use or documentation bias, and future prospective studies should collect structured sleep and medication/supplement data to clarify directionality and mechanisms. Finally, recent use of oral antibiotics that potentially influence coagulation by reducing the gut flora responsible for endogenous vitamin K production can be relevant, particularly in the case of β-lactam antibiotics such as cefoperazone [27,28].

These results emphasize the multifactorial nature of antibiotic-associated coagulation derangement and the relevance of nutritional, microbiological, hepatotoxic, and geriatric comorbidities in clinical risk evaluations. Our results are in line with the prior literature, which recommended cautious use of the cefoperazone–sulbactam combination in patients with advanced age and poor nutrition due to the higher risk of coagulopathy [2,10,29]. Data from previous pharmacovigilance studies and case series have reported the potential for cefoperazone to inhibit the synthesis of vitamin K–dependent clotting factors, possibly associated with inhibition via the N-methylthiotetrazole (NMTT) side chain or inhibition of intestinal microbiota. However, most previous studies lacked structured modeling or did not provide quantified risk in different clinical subsets [2,30].

To the best of our knowledge, the present study is the most systematic and extensive exploration of different ML models for coagulation dysfunction specifically caused by cefoperazone–sulbactam in the geriatric inpatient population. Although machine learning has been used to predict surgical or anticoagulant-related bleeding risk in previous studies, our study examines a more specific, medication-related adverse event in a real-world, non-interventional setting. Furthermore, the application of SHAP values allows interpretability beyond conventional statistical models, thus permitting clinicians to gain insights into the contribution of each variable to risk profiles on an individual level.

The recognition of insomnia as a possible predictor is also congruent with a growing body of literature supporting an association between sleep disturbance, systemic inflammation, and vascular endothelial dysfunction, albeit a bridge still to be strategically crossed within the backdrop of antibiotic-associated coagulopathy [31,32]. Therefore, our findings add to the existing knowledge that combines typical clinical judgment with algorithmic expertise. Our model is of great value in preventing cefoperazone–sulbactam therapy-related injury in older people. Through the incorporation of readily available clinical variables into a predictive model, clinicians can identify patients at increased risk of coagulopathy to intervene before the development of clinically apparent hemorrhage. This is especially important in geriatric medicine, where minor laboratory alterations may signify early stages of clinical deterioration and where early intervention could avoid cascades ending in major morbidity.

Crucially, the LightGBM model was not only more accurate but also remained sensitive for ward-based screening of high-risk inpatients (e.g., geriatric and intensive care settings). Associated SHAP-based explanations deliver actionable insight by identifying which patient attributes drive the risk the most so that clinicians can build trust and use it for shared decision-making [33,34,35].

While logistic regression supports a simple, transparent nomogram suitable for bedside scoring, LightGBM achieved improved discrimination and sensitivity. To preserve clinical usability, we combined LightGBM with SHAP-based explanations and a web-based calculator, providing both performance and accessibility. More importantly, the construction of an easy-to-use nomogram and the establishment of a web-based calculator based on Shiny directly convert this model to an easily applied clinical reference. They can be used for bedside risk assessment and to assist in management decisions, including early vitamin K supplementation, closer monitoring of hemostasis, and the selection of a different antibiotic in susceptible patients. As the clinical implementation of machine learning methods continues to expand, such interpretability and accessibility of models will be important for their real-world use.

There are several notable strengths to this study. It is one of the first papers to compare a large number of machine learning algorithms with traditional logistic regression to predict cefoperazone–sulbactam-associated coagulopathy in elderly inpatients. The two-center design (Fuxing Hospital and Xuanwu Hospital of Capital Medical University) increases the heterogeneity and generalizability of the data. The incorporation of real-world collected variables increases the generalizability of the model, and the SHAP analysis and nomogram development increase interpretability. Finally, the introduction of a web-based risk calculator highlights the translation of the study to clinical practice.

Nonetheless, limitations remain. Although this is a multi-institutional study, it was conducted at one metropolitan area and was performed in a retrospective fashion that can introduce selection and documentation biases. Some possibly related predictors, such as serum concentrations of vitamin K, more detailed liver function tests, or the composition of gut microbiota, were not measured or could not be included in the model. Some laboratory parameters with high biological relevance—particularly coagulation factor VII (Factor VII) and other vitamin K–dependent factors—were not routinely available in this retrospective dataset and therefore could not be incorporated. Given the short half-life of Factor VII, future prospective work should prioritize serial assessment of Factor VII (and related factors) to better characterize early functional changes. In contrast, vitamin K concentration alone may not fully reflect downstream coagulation factor activity and may provide limited mechanistic resolution without concurrent functional factor measurements. Although internal validation was performed, the absence of external validation limits generalizability; external testing in independent cohorts is a key priority for future work.

Based on the current study, future research should include validation of the proposed model in a prospective, multicenter design to ensure its applicability across different patient populations and healthcare systems. The addition of time-dependent data, such as time-variant coagulation parameters or nutritional markers, might increase predictive granularity and responsiveness of the model. Moreover, inclusion of laboratory biomarkers not measured in the present study, such as serum levels of vitamin K, liver function indices (i.e., factor V, INR), and inflammation-induced cytokines, would increase biological specificity.

An important direction is to implement the model into an electronic health record (EHR) system for real-time clinical decision support. This could enable automated risk alerts at the time of prescription or antibiotic administration for timely preventive interventions (e.g., coagulation monitoring, vitamin K supplementation, or reconsideration of antibiotic choice). Lastly, it would be interesting to investigate an adaptive or ensemble modeling approach with clinician feedback that could support iterative learning and continuous improvement of the models in practice.

## 5. Conclusions

This research effectively established and verified a machine learning–powered predictive model for early detection of coagulation disorders among elderly inpatients undergoing treatment with cefoperazone–sulbactam. Of the ten algorithms compared, the predictive capability and clinical utility of the LightGBM model were the best. Responsibility of main risk factors, including older age, hypoalbuminemia, parenteral alimentation, impaired sleep, and oral antibiotic medication, was determined on the results of logistic regression analysis and explained using SHAP analysis.

Between the model accuracy, interpretability, and accessibility via the web-based instrument, this work provides an applicable method for early risk stratification and personalized clinical decision support. These results emphasize the potential of machine learning to improve monitoring of antibiotic safety in vulnerable populations, and suggest that future model refinement and external validation will be required in wider healthcare contexts.

## Figures and Tables

**Table 1:** Characteristics of included patients.

Demographic Characteristics	Desc	No Coagulation Abnormalities (*n* = 335)	Coagulation Abnormalities (*n* = 150)	*p*
Age	≥75	142 (42.4%)	92 (61.3%)	<0.001
	60–75	193 (57.6%)	58 (38.7%)	
Gender	Female	102 (30.4%)	53 (35.3%)	0.337
	Male	233 (69.6%)	97 (64.7%)	
History_of_intravenous_antibiotic_use	No	243 (72.5%)	89 (59.3%)	0.005
	Yes	92 (27.5%)	61 (40.7%)	
History_of_oral_antibiotics	No	206 (61.5%)	65 (43.3%)	<0.001
	Yes	129 (38.5%)	85 (56.7%)	
feeding_state	Normal_Diet	180 (53.7%)	56 (37.3%)	<0.001
	Total_Gastrointestinal_Parenteral_Nutrition	28 (8.4%)	27 (18%)	
	Transnasal_Gastric_Tube_Diet	127 (37.9%)	67 (44.7%)	
Hepatic_insufficiency	No	318 (94.9%)	145 (96.7%)	0.538
	Yes	17 (5.1%)	5 (3.3%)	
Renal_insufficiency	No	242 (72.2%)	108 (72%)	1
	Yes	93 (27.8%)	42 (28%)	
antiplatelet	No	230 (68.7%)	85 (56.7%)	0.014
	Yes	105 (31.3%)	65 (43.3%)	
hypoproteinemia	No	112 (33.4%)	18 (12%)	<0.001
	Yes	223 (66.6%)	132 (88%)	
Hyperlipidemia	No	192 (57.3%)	104 (69.3%)	0.016
	Yes	143 (42.7%)	46 (30.7%)	
Cardiovascular_disease	No	211 (63%)	80 (53.3%)	0.057
	Yes	124 (37%)	70 (46.7%)	
Marital_status	married	159 (47.5%)	74 (49.3%)	0.777
	other	176 (52.5%)	76 (50.7%)	
smoking	No	269 (80.3%)	116 (77.3%)	0.532
	Yes	66 (19.7%)	34 (22.7%)	
drinking	No	191 (57%)	92 (61.3%)	0.428
	Yes	144 (43%)	58 (38.7%)	
insomnia	No	104 (31%)	26 (17.3%)	0.002
	Yes	231 (69%)	124 (82.7%)	
Hypertension	No	170 (50.7%)	84 (56%)	0.331
	Yes	165 (49.3%)	66 (44%)	
Diabetes	No	210 (62.7%)	95 (63.3%)	0.972
	Yes	125 (37.3%)	55 (36.7%)	
BMI	Mean ± SD	29.2 ± 4.0	28.7 ± 4.3	0.205

**Table 2:** Training set univariate and multivariate logistic regression results.

Name	Desc	No Coagulation Abnormalities (*n* = 235)	Coagulation Abnormalities (*n* = 105)	OR (Univariable)	OR (Multivariable)
Age	60–75	136 (57.9%)	41 (39%)		
	≥75	99 (42.1%)	64 (61%)	2.14 (1.34–3.43, *p* = 0.002)	2.13 (1.27–3.56, *p* = 0.004)
Gender	Female	71 (30.2%)	35 (33.3%)		
	Male	164 (69.8%)	70 (66.7%)	0.87 (0.53–1.42, *p* = 0.566)	
History_of_intravenous_antibiotic_use	No	174 (74%)	60 (57.1%)		
	Yes	61 (26%)	45 (42.9%)	2.14 (1.32–3.47, *p* = 0.002)	1.58 (0.29–8.71, *p* = 0.602)
History_of_oral_antibiotics	No	146 (62.1%)	44 (41.9%)		
	Yes	89 (37.9%)	61 (58.1%)	2.27 (1.42–3.63, *p* < 0.001)	1.90 (1.13–3.19, *p* = 0.015)
feeding_state	Normal_Diet	129 (54.9%)	37 (35.2%)		
	Transnasal_Gastric_Tube_Diet	88 (37.4%)	47 (44.8%)	1.86 (1.12–3.10, *p* = 0.017)	1.35 (0.77–2.35, *p* = 0.290)
	Total_Gastrointestinal_Parenteral_Nutrition	18 (7.7%)	21 (20%)	4.07 (1.96–8.42, *p* < 0.001)	3.82 (1.71–8.53, *p* = 0.001)
Hepatic_insufficiency	No	221 (94%)	100 (95.2%)		
	Yes	14 (6%)	5 (4.8%)	0.79 (0.28–2.25, *p* = 0.658)	
Renal_insufficiency	No	167 (71.1%)	76 (72.4%)		
	Yes	68 (28.9%)	29 (27.6%)	0.94 (0.56–1.56, *p* = 0.804)	
antiplatelet	No	167 (71.1%)	58 (55.2%)		
	Yes	68 (28.9%)	47 (44.8%)	1.99 (1.24–3.21, *p* = 0.005)	3.11 (0.36–27.01, *p* = 0.304)
hypoproteinemia	No	73 (31.1%)	17 (16.2%)		
	Yes	162 (68.9%)	88 (83.8%)	2.33 (1.30–4.20, *p* = 0.005)	3.19 (1.64–6.19, *p* < 0.001)
Hyperlipidemia	No	134 (57%)	71 (67.6%)		
	Yes	101 (43%)	34 (32.4%)	0.64 (0.39–1.03, *p* = 0.066)	
Cardiovascular_disease	No	154 (65.5%)	55 (52.4%)		
	Yes	81 (34.5%)	50 (47.6%)	1.73 (1.08–2.76, *p* = 0.022)	0.43 (0.11–1.72, *p* = 0.231)
Marital_status	other	114 (48.5%)	51 (48.6%)		
	married	121 (51.5%)	54 (51.4%)	1.00 (0.63–1.58, *p* = 0.992)	
smoking	No	187 (79.6%)	84 (80%)		
	Yes	48 (20.4%)	21 (20%)	0.97 (0.55–1.73, *p* = 0.928)	
drinking	No	135 (57.4%)	66 (62.9%)		
	Yes	100 (42.6%)	39 (37.1%)	0.80 (0.50–1.28, *p* = 0.349)	
insomnia	No	78 (33.2%)	18 (17.1%)		
	Yes	157 (66.8%)	87 (82.9%)	2.40 (1.35–4.27, *p* = 0.003)	2.94 (1.56–5.52, *p* < 0.001)
Hypertension	No	123 (52.3%)	59 (56.2%)		
	Yes	112 (47.7%)	46 (43.8%)	0.86 (0.54–1.36, *p* = 0.511)	
Diabetes	No	147 (62.6%)	64 (61%)		
	Yes	88 (37.4%)	41 (39%)	1.07 (0.67–1.72, *p* = 0.779)	
BMI	Mean ± SD	29.3 ± 4.1	28.8 ± 4.4	0.97 (0.92–1.03, *p* = 0.305)	

**Table 3:** Evaluation metrics for training and test sets.

	Model	Threshold	Accuracy	Sensitivity	Specificity	Precision	F1
Training set	Logistic	0.307029239116951	0.676	0.676	0.677	0.483	0.563
Training set	SVM	0.346979348147734	0.691	0.59	0.736	0.5	0.541
Training set	GBM	0.297495257362656	0.682	0.686	0.681	0.49	0.571
Training set	NeuralNetwork	0.317381615064435	0.671	0.676	0.668	0.477	0.559
Training set	RandomForest	0.5	0.741	0.381	0.902	0.635	0.476
Training set	Xgboost	0.290711522102356	0.682	0.724	0.664	0.49	0.585
Training set	KNN	0.305057131154246	0.665	0.695	0.651	0.471	0.562
Training set	Adaboost	0.384467935585538	0.682	0.686	0.681	0.49	0.571
Training set	LightGBM	0.316962880667173	0.688	0.714	0.677	0.497	0.586
Training set	CatBoost	0.576578764055355	0.644	0.714	0.613	0.452	0.554
Test set	Logistic	0.307029239116951	0.655	0.756	0.61	0.466	0.576
Test set	SVM	0.370070341530869	0.676	0.6	0.71	0.482	0.535
Test set	GBM	0.303425665153187	0.683	0.733	0.66	0.493	0.589
Test set	NeuralNetwork	0.317381615064435	0.655	0.756	0.61	0.466	0.576
Test set	RandomForest	0.5	0.724	0.333	0.9	0.6	0.429
Test set	Xgboost	0.321540772914886	0.676	0.756	0.64	0.486	0.591
Test set	KNN	0.412975352325084	0.676	0.556	0.73	0.481	0.515
Test set	Adaboost	0.374676276034012	0.676	0.756	0.64	0.486	0.591
Test set	LightGBM	0.220519577198697	0.6	0.978	0.43	0.436	0.603
Test set	CatBoost	0.576482981624328	0.634	0.844	0.54	0.452	0.589

## Data Availability

The de-identified patient-level data and the codes used in the current study are available from the corresponding author upon reasonable request. The data are not publicly available due to privacy and ethical restrictions related to patient clinical information.

## Abbreviations

The following abbreviations are used in this manuscript:

PT	prothrombin time
APTT	activated partial thromboplastin time
SHAP	SHapley Additive exPlanations
ORs	odds ratios
CIs	confidence intervals
DCA	decision curve analysis
AUC	area under the curve
